# Synthesis of DNA/RNA and Their Analogs via Phosphoramidite and H-Phosphonate Chemistries 

**DOI:** 10.3390/molecules181114268

**Published:** 2013-11-18

**Authors:** Subhadeep Roy, Marvin Caruthers

**Affiliations:** Department of Chemistry and Biochemistry, University of Colorado, Boulder, CO 80309, USA; E-Mail: Marvin.Caruthers@Colorado.edu

**Keywords:** DNA, RNA, phosphoramidites, H-phosphonates, boranephosphonates, metallophosphonates, boranealkylphosphines

## Abstract

The chemical synthesis of DNA and RNA is universally carried out using nucleoside phosphoramidites or H-phosphonates as synthons. This review focuses on the phosphorus chemistry behind these synthons and how it has been developed to generate procedures whereby yields per condensation approach 100% with very few side products. Additionally the synthesis and properties of certain DNA and RNA analogs that are modified at phosphorus will also be discussed. These analogs include boranephosphonates, metallophosphonates, and alkylboranephosphines.

## 1. Introduction

The ability to chemically synthesize 2'-deoxyoligonucleotides and oligoribonucleotides (oligonucleotides) in high yield and purity has revolutionized biochemical research as well as enabled a large number of other technologies such as DNA/RNA based therapeutics, DNA forensics, and high-throughput sequencing. The chemical structure of DNA/RNA consists of purines and pyrimidines attached to 2'-deoxyribose or ribose sugars. These 2'-deoxyribonucleosides or ribonucleosides are then joined via phosphodiester linkages. Thus phosphorus chemistry is central to the development of synthetic methods for preparing these important biological molecules and for achieving the high yield, high-throughput synthesis of oligonucleotides. 

In this review we will focus on one aspect of organophosphorus chemistry, namely the reactivity of P (III) centers that enables the synthesis of DNA/RNA. We will describe these developments by first reviewing the work of Robert Letsinger and co-workers as their research led to the phosphoramidite approach in our laboratory. We will also discuss the H-phosphonate methodology that is an alternative P (III) chemistry for the synthesis of oligonucleotides. Both approaches have been used to prepare a plethora of nucleic acid analogs modified at phosphorus. This is a vast field of research in itself and a comprehensive description of all the different derivatives is outside the scope of this review. We will instead discuss a recent and promising class of analogs in which a P (III) center is bonded to a Lewis acid such as borane and forms stable internucleotide linkages. 

## 2. Synthesis of DNA Using Chlorophosphites and Tetrazoylphosphites

Development of the phosphoramidite method of DNA synthesis was based upon two observations. One was that chloro- and dichlorophosphites react rapidly with the 3'-hydroxyl group of a 2'-deoxynucleoside, whereas the corresponding phosphorochloridates require several hours at room temperature [1]. Yields were also much higher with chloro and dichlorophosphites. Thus the possibility existed that DNA could be prepared rapidly and in high yield from activated phosphites. The other observation was that Robert Letsinger’s laboratory had used dichlorophosphites to synthesize a 2'-deoxythymidine pentanucleotide. Stepwise yields for addition of each mononucleotide were 69%–82%. Most encouraging was the observation that reaction times were only 5 minutes in comparison to hours for earlier approaches. 

These observations led us to explore the use of 2'-deoxynucleoside P (III) derivatives for synthesizing 2'-deoxyoligonucleotides on polymeric supports [2,3]. For several reasons, our research was predicated on the use of HPLC-grade silica (also known as controlled pore glass (CPG)) as a support. One was that all previous research utilizing organic polymers, such as polystyrene, to synthesize DNA [4] had failed in part because organic supports readily adsorbed the synthons and other reagents. Thus removal of these materials after each condensation step was difficult. This problem, when coupled with the knowledge that HPLC-grade silica had been designed for efficient mass transfer, dictated its investigation. Our expectation was that both solvents and reagents could be removed rapidly and completely from the matrix. This was observed in our early work. Silica was also expected to be chemically inert towards all the reagents we contemplated using and was a rigid, non-swelling matrix in common organic solvents. Therefore, it could be packed into a column and reactants merely pumped through the column. 

**Scheme 1:**
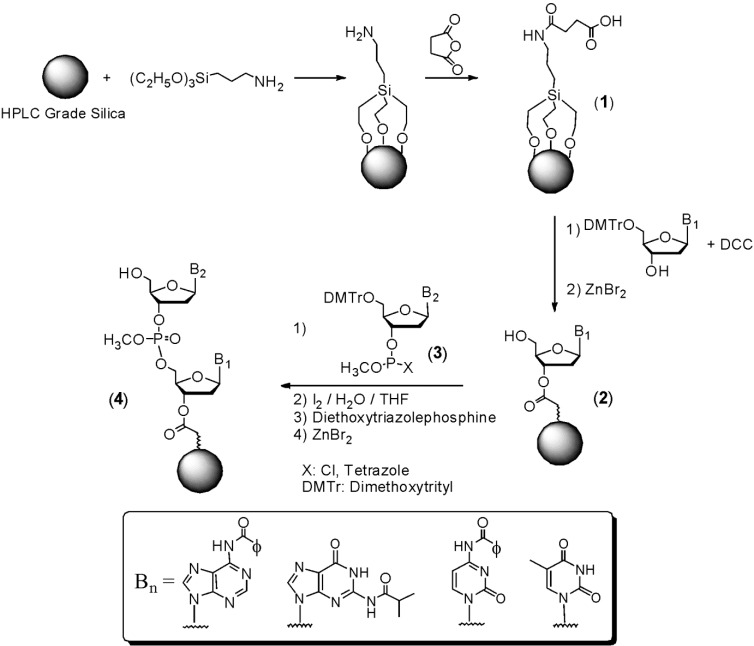
DNA Synthesis on HPLC Silica.

The initial approach we developed is outlined in Scheme 1. The first step was activating the silica matrix by attaching (3-aminopropyl)triethoxysilane to silica and then treating the product of this reaction with succinic anhydride in order to generate **1**. The next step was condensing 5'-dimethoxytrityl-2'-deoxythymidine to the support using dicyclohexylcarbodiimide (DCC) to activate the carboxylic acid. After removal of the 5'-dimethoxytrityl group with ZnBr_2_ to yield **2**, condensation with a 2'-deoxynucleoside 3'-phosphite **3** was carried out. Of the synthons we examined, the most reactive while generating the fewest side-products, were the 5'-dimethoxytrityl 3'-methyltetrazoyl phosphites. Using these synthons, 95% yields per condensation were observed during synthesis. After oxidation of the phosphite to phosphate with aqueous iodine, capping with diethoxytriazolephosphine to remove any unreacted intermediates, and removal of the dimethoxytrityl group with ZnBr_2_, the product **4** of this reaction sequence could be extended by repetitive use of this cycle in order to generate a 2'-deoxyoligonucleotide. Finally after completion of oligonucleotide synthesis, methyl groups were removed from phosphates using thiophenol. This oligomer was then cleaved from the support and base protecting groups (*N*-isobutyrylguanine, *N*-benzoylcytosine and *N*-benzoyladenine) removed with ammonium hydroxide. By measuring the amount of dimethoxytrityl cation released following synthesis of a 12mer, the overall yield was determined to be 55%, which was unprecedented at that time in the nucleic acid field.

During the course of this work, we also developed a semiautomatic machine where one cycle of synthesis on a silica column, including the use of all reagents and solvents, could be programmed and completed automatically. The operator then just added the next appropriately protected 2'-deoxy-nucleoside-3'-phosphite to the column and initiated another synthesis cycle. Although more successful than any previous method, it was still far from acceptable. The main problem was that the 2'-deoxy-nucleoside 3'-tetrazoylphosphites had to be prepared at −78 °C and preferably used the same day. The most significant outcome from this research that continues to the present, was the successful development of silica as a matrix for DNA synthesis.

## 3. The Phosphoramidite Approach for Synthesis of DNA and RNA

Our discovery of phosphoramidites as synthons was initiated with a study aimed at activating aminophosphines. The strategy was to activate 5'-dimethoxytrityl 2'-deoxynucleoside 3'-amino-phosphines via insertion of CO_2_, CS_2_ or COS to form mixed anhydrides [5,6] that we posited would condense with a 2'-deoxynucleoside. In our hands, this strategy was not successful. However during the attempted synthesis of the aminophosphines by reacting 5'-*O*-dimethoxytrityl-2'-deoxythymidine with N,N-dimethylaminomethoxychlorophosphine in pyridine, we observed the appearance of a new dimethoxytrityl positive spot on TLC (thin-layer chromatography) that moved similar to a dinucleotide. Based upon the previous literature on the acid activation of aminophosphines [7], we reasoned that the initial product, 5'-dimethoxytrityl 2'-deoxythymidine 3'-*N*,*N*-dimethylamino-methoxyphosphine (a phosphoramidite), was activated by pyridine hydrochloride that was produced *in situ* during the reaction. The protonated 5'-dimethoxytrityl 2'-deoxythymidine 3'-*O*-methoxy-*N*,*N*-dimethylammonium phosphine would then react with the free 3'-hydroxyl of excess 5'-dimethoxytrityl 2'-deoxythymidine present in the reaction mixture to form *bis*(5'-dimethoxytrityl 2'-deoxythymidine 3'-*O*) methylphosphite. Subsequently by repeating this reaction in the presence of a stronger base (Hunig’s base) in the reaction mixture in order to remove the acid produced, we were able to isolate the desired phosphoramidites. This allowed us to confirm that phosphoramidites could indeed be activated with ease towards nucleophilic substitution using a number of weak acids. We then proceeded to combine this discovery with the use of CPG as a solid support to develop a high yielding method for oligonucleotide synthesis that has remained essentially unmodified since our original report [8].

**Scheme 2:**
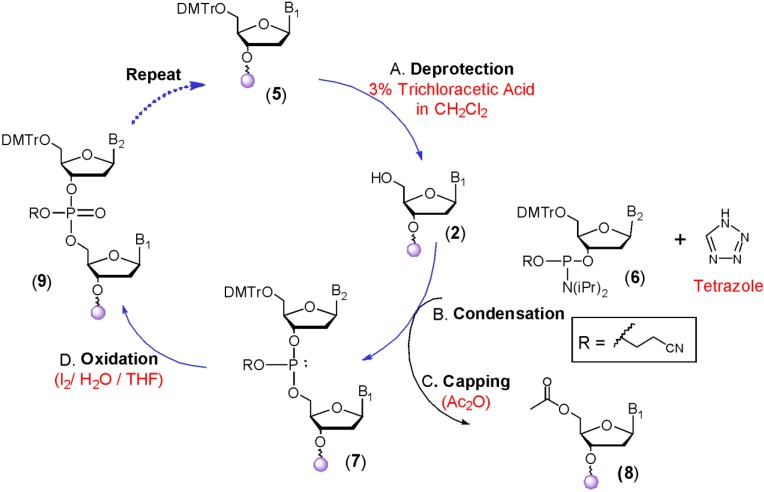
The Phosphoramidite Approach for Oligonucleotide Synthesis.

The synthesis of DNA and RNA using the phosphoramidite approach is depicted in Scheme 2. The synthetic cycle begins with the removal of 5'-DMTr from **5** by treatment with a solution of 3% trichloroacetic acid in dichloromethane to yield **2**. Condensation of a 5'-dimethoxytrityl-2'-deoxynucleoside 3'-phosphoramidite **6** with **2** is then completed using tetrazole as an activator to produce the phosphite triester **7**. The most commonly used phosphoramidites contain a diisopropyl-amino group. The steric bulk of this group was found to provide an ideal balance between stability during preparation of these synthons and ease of activation [9]. Although many activators have been proposed, tetrazole remains a popular choice as it can be obtained as a solid by sublimation and kept stably as an anhydrous solution on the DNA synthesis machine for long periods of time. 

The next step is capping of unreacted 5'-hydroxyl groups using acetic anhydride activated by N-methylimidazole in pyridine (Step C). This is important as it prevents any unreacted oligonucleotides from continuing to grow. Failing to do this leads to a final product mixture whereby the major oligonucleotide impurities are hard to remove because they are shorter by only a few nucleotides. For the synthesis of DNA (≥150 mers) on glass chips using inkjet printers, the capping step was found to be unnecessary [10]. This likely resulted from surface effects that allow even better condensation yields than possible with traditional CPG supports. The final step is oxidation of the phosphite triester to phosphate **9** using aqueous iodine. The entire sequence can then be repeated iteratively to obtain the desired oligomer. For the synthesis of various analogs such as phosphorothioates and phosphoramidates, oxidation can alternatively be carried out using a sulfurizing agent or iodine and amines respectively. 

At this stage, the oligomer can be hydrolyzed from the support using an aqueous solution of ammonia which also removes the amide groups used to protect the exocylic amines of cytosine, guanine and adenine as well as the cyanoethyl protection [11] on phosphate. The oligomers can then be purified by reverse phase HPLC or gel electrophoresis. 

**Scheme 3:**
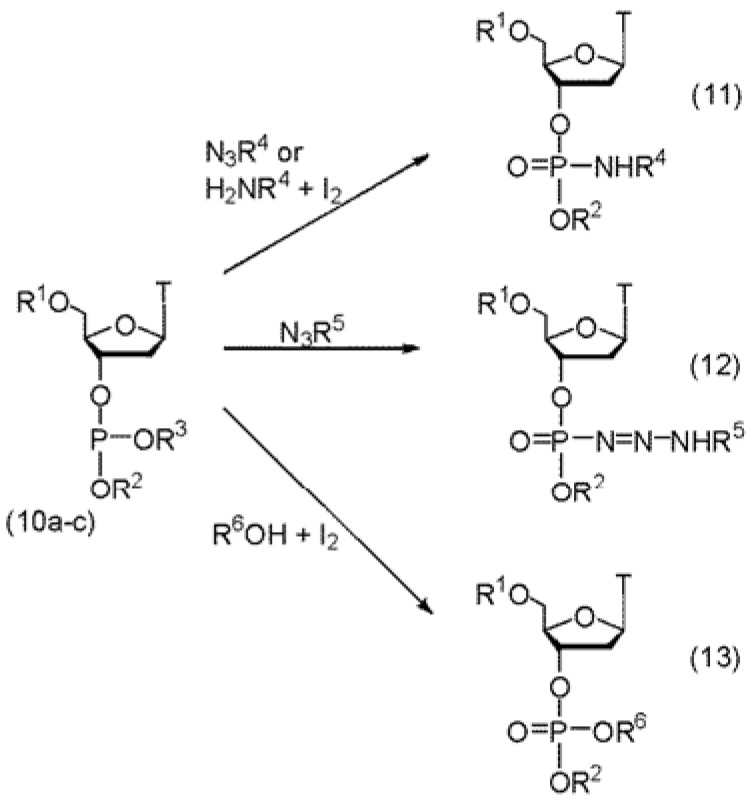
(**a**–**c**): R^1^ = 4,4'-dimethoxytrityl, R^2^ = 3'-acetylthymidylyl; a: R^3^ = 2-cyano-1,1-dimethylethyl, R^4^ = *n*-butyl; b: R^2^ = 3'-acetylthymidylyl, R^5^ = 3-(*N*-ethylcarbazoyl), R^3^ = *o*-methylbenzyl; c: R^2^ = methyl, R^3^ = 2-cyano-1,1-dimethylethyl, R^6^ = H, alkyl, or aryl.

During an exploration of various phosphorus protecting groups, we observed that *o-*methylbenzyl was removed during the iodine oxidation step [12]. Moreover a deoxyoligonucleotide 20 in length, as prepared with this protecting group, gave a very high yield of a homogeneous product. At the same time, van Boom and co-workers published similar results with the 2-cyano-1,1-dimethylethyl phosphorus protecting group [13]. These observations led us to propose that alkyl deoxynucleoside phosphites (**10a**–**c**, Scheme 3), having alkyl groups that stabilize the S_N_1 character of the second stage of an Arbuzov reaction, upon reaction with most electrophiles, would lead to elimination of the alkyl protecting group with concomitant formation of the phosphoryl bond. Thus upon oxidation with iodine (the electrophile), the tertiary alkyl protecting group would be eliminated and generate the phosphoroiodate. Nucleophilic substitution of iodine would yield various desired compounds. For example if the reaction were performed in aqueous iodine, the product would be the natural, internucleotide phosphate diester linkage (compound **13**, R^6^ = H). If however oxidation were to take place under anhydrous conditions in the presence of amines, azides or alcohols then the appropriate phosphoramidates **11**, phosphoroazoamidates **12**, or phosphate triesters **13** (R^6^ = alkyl or aryl) would be the final products. In contrast if these phosphites were oxidized with *tert-*butylhydroperoxide or sulfur, the product was the dinucleotide phosphate triester or thiophosphate triester respectively. Removal of the 2-cyano-1,1-dimethylethyl protecting group with base would then generate the phosphate diester or phosphorothioate diester. Thus this approach, depending upon oxidation conditions for each synthesis cycle, can be used to introduce different analogs selectively throughout an oligonucleotide. 

## 4. The H-Phosphonate Approach for Synthesis of DNA and RNA

H-Phosphonates are a distinct category of tervalent P (III) compounds that contain a phosphoryl group (P=O) and a hydrogen atom bonded to the phosphorous center (e.g., **18**). H-Phosphonates have a tetrahedral geometry that is similar to P (V) compounds. The phosphorus atom in H-phosphonates is electrophillic and lacks a lone pair of electrons. Therefore it is much more resistant towards oxidation under ambient conditions than most P (III) compounds. At the same time, upon activation, H-Phosphonate monoesters can be induced to undergo nucleophillic substitutions efficiently. This combination of properties has made them attractive synthons for preparing oligonucleotides.

**Scheme 4:**
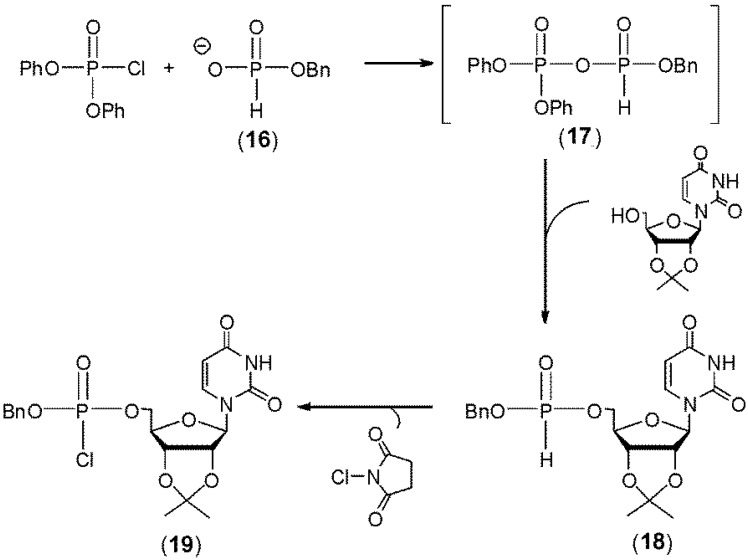
Mixed Anhydride Activation of H-Phosphonates.

H-Phosphonates were first used in nucleotide chemistry by Sir Alexander Todd [14,15]. They demonstrated that treatment of benzyl H-phosphonate monoester **16** with diphenyl phosphorochloridate (Scheme 4) led to the putative formation of the corresponding activated mixed anhydride **17**. This compound was reacted *in situ* with the 5'-hydroxyl of a 2',3'-isopropylidine nucleoside to produce the H-phosphonate diester **18** that was in turn oxidized by *N*-chlorosuccinimide to the phosphorochloridate diester **19**. They later adapted this methodology for the first chemical synthesis of a natural 5'-3' linked dinucleotide [16]. Ogilvie and Nemer in 1980 [17] as well as Kume *et al*. in 1984 [18] next demonstrated the synthesis of dinucleotides containing H-phosphonate internucleotide linkages. However the use of H-phosphonates as synthons for oligonucleotide synthesis was not developed till three decades after Todd’s initial reports, when Garegg *et al.* [19] and later Froehler and Matteucci [20] described the synthesis of 2'-deoxynucleoside H-phosphonate diesters by activating 2'-deoxynucleoside H-phosphonate monoesters with several reagents such as 2,4,6-tri- isopropylbenzenesulfonylchloride, *N*,*N*-*bis*(2-oxo-3-oxazolidinyl)-phosphorodiamide chloride, diphenyl-chlorophosphate and pivaloyl chloride. 

**Scheme 5:**
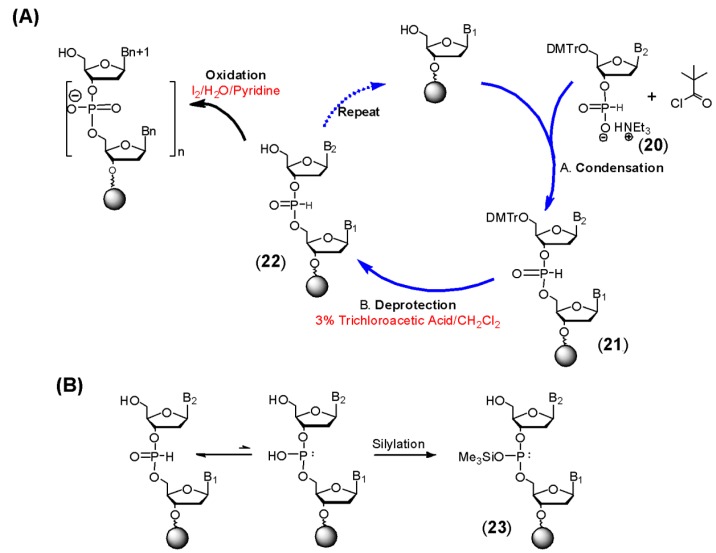
The H-Phosphonate Approach to DNA/RNA Synthesis.

Froehler *et al.* [21] and Garegg *et al.* [22,23] next demonstrated the use of this method for the solid phase synthesis of oligonucleotides using 2'-deoxy and ribonucleoside H-phosphonate monoesters as synthons. The general method is shown in Scheme 5A. Following acid-mediated removal of the 5'-dimethoxytrityl protecting group, condensation is carried out by addition of an appropriately protected 2'-deoxy or ribonucleoside H-phosphonate monoester **20** and an activating agent—typically pivaloyl chloride or adamantoyl chloride. Reaction of the acid chloride with the H-phosphonate monoester produces the corresponding mixed phosphonocarboxylic anhydride. This mixed anhydride undergoes a nucleophilic attack at the phosphorous by the 5'- hydroxyl of the nucleoside joined to a polymer support. The product of this reaction is the H-phosphonate diester **21**. Unlike the phosphite triester **7**, this linkage is stable to the acidic conditions (3% trichloroacetic acid in dichloromethane) required for removal of the 5'-dimethoxytrityl group. Thus it is unnecessary to carry out oxidation at every cycle. Chain elongation therefore consists of two steps. Oxidation to the phosphodiester with aqueous iodine is completed following oligonucleotide synthesis. Alternatively oxidation can be carried out under non-aqueous conditions using various reagents which has enabled the synthesis of a number of modified oligonucleotides such as phosphoramidates [24], phosphorothioates [25], and phosphoro-selenoates [26]. After the oxidation step, aqueous ammonia is used to remove nucleobase amide protecting groups and to cleave the oligonucleotide from the support.

The H-phosphonate method is particularly well suited for RNA synthesis as some of the problems associated with this approach, such as double activation and phosphorus acylation, are ameliorated when using ribonucleosides having a 2'-protecting group [27]. Coupling of ribonucleoside H-phosphonates on a solid support when compared to coupling ribonucleoside phosphoramidites is less sensitive to steric effects that arise due to the 2'-protecting group. RNA molecules up to 50 to 60 nucleotides in length can be made in high yields by this method. 

An additional attraction of this chemistry for synthesis of certain DNA or RNA derivatives stems from the fact that the H-phosphonate diester exists in equilibrium with its phosphite form (Scheme 5B). Ordinarily the H-phosphonate is more stable and the predominant species. However silylation can be used to trap the phosphite triester form **23**, which creates a nucleophilic phosphorus center having a lone pair of electrons. This phosphite triester can then be treated with electrophiles to allow synthesis of various phosphorus analogs. As the silylation procedure can be performed at the end of the synthetic cycles, repetitive exposure of the desired derivative to the acidic deprotection solution is avoided. This is an important advantage when preparing acid sensitive analogs. However a limitation of this protocol is that only uniformly modified oligomers can be produced. H-Phosphonate diesters also have stable stereochemical configurations and undergo oxidation or conversion to phosphite triesters (as in Scheme 5B) in a stereospecific manner [28]. This has been exploited as a method for the synthesis of stereodefined DNA derivatives. 

H-Phosphonates offer a useful alternative to the phosphoramidite method particularly for the synthesis of RNA and acid labile oligonucleotide analogs. Although phosphoramidites continue to be much more widely adopted because of higher stepwise yields and fewer side-products, it should be noted that at present H-phosphonates are the only other commercially available DNA/RNA synthons. It is expected that H-phosphonate chemistry will continue to be useful for the preparation of certain novel DNA derivatives.

## 5. DNA Containing Stable Complexes of P (III) with Lewis Acids

**Figure 1:**
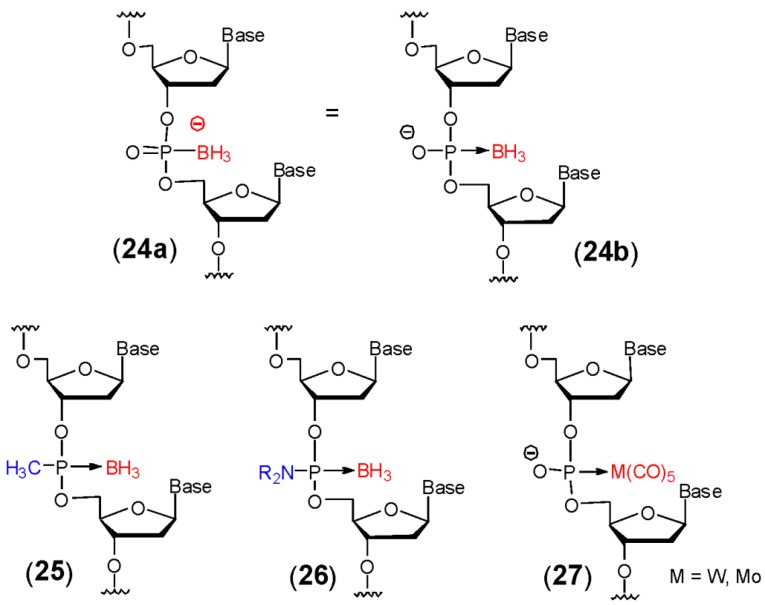
Lewis Acid—Phosphorous Analogs of DNA.

The presence of a lone pair of electrons on the phosphorous atom in P (III) compounds allows it to form stable complexes with Lewis acids. Such complexation stabilizes the phosphorous towards oxidation or attack by electrophiles. Barbara Ramsay Shaw and co-workers were the first to exploit this bonding scheme in their synthesis of 2'-deoxythymidylyl-(3'-5')-2'-deoxythymidine which contained a hydrolytically stable boranephosphonate (also referred to as boranophosphate in the literature) linkage **24** (Figure 1) [29,30]. These compounds are conventionally described as having one of the non-bridging phosphate oxygens replaced by borane (compound **24a**). This depiction emphasizes that the stability of these compounds is similar to phosphate diesters. On the other hand this linkage can also be described as a P (III) compound complexed with a borane group (compound **24b**). This representation ties these analogs to a wider category of similar compounds and helps to illustrate the fact that bonding of this type can be a general approach to creating varied backbone structures with engineered properties. For example we have reported that methyphosphine diesters complexed with borane (compound **25**) [31] yield a stable structure that is neutral as opposed to the negatively charged boranephosphonate. Oligomers containing these neutral linkages demonstrate enhanced cellular uptake. This approach can be extended to include a number of substituents including amines (compound **26**) and acetate (*vide infra*) with each having unique and tailored properties. In addition to borane, other Lewis acids can also be used as demonstrated by the synthesis of dinucleotides containing a P (III) center complexed to pentacarbonyltungstate(-1) and pentacarbonyl-molybdate(-1) (**27**) [32,33]. These metallophosphonate derivatives should prove to be useful molecules for the construction of DNA based circuits and metallic nanostructures. Because extensive research on the chemical and biochemical properties of boranephosphonate DNA and RNA (bpDNA or bpRNA) has already been completed, the primary emphasis in the following section is on bpDNA with only brief mention of the synthetic methods used to prepare the other analogs shown in Figure 1.

Although the first report of dithymidine having a boranephosphonate diester linkage was published more than twenty years ago, the lack of high yielding chemical methods till recently has severely limited the applicability of these molecules. However in the interim, the Ramsay-Shaw laboratory developed an elegant enzymatic method for synthesizing bpDNA and bpRNA. Their procedure used 2'-deoxynucleoside or ribonucleoside triphosphates that contained a borane moiety at the α phosphorous. When these triphosphates were used with certain polymerases and an appropriate primer/template, bpDNA or bpRNA could readily be prepared [34,35,36,37]. Enzymatically synthesized bpDNA (or bpRNA) enabled early studies that were important in demonstrating the biochemical potential of boranephosphonate oligonucleotides. Unfortunately enzymatic synthesis was not only expensive and low yielding but, because only one of the dNTPs could be replaced by its corresponding borane derivative during any one synthesis, the flexibility of positioning and density of boranephosphonate linkages within an oligomer was severely restricted. However it should be noted that an advantage of enzymatic synthesis was that polymerases recognize only one of the two enantiomers of α-P-borano dNTPs. Thus diastereomerically pure oligomers were produced by enzymatic synthesis whereas chemical methods generate mixtures of diastereomers.

Conceptually, the chemical synthesis of boranephosphonates simply requires substituting oxidation in the synthesis cycle with boronation. Chemically this means that a BH_3_ group is exchanged between a labile borane complex (such as THF-borane) and the phosphite triester (e.g., **7**, Scheme 2). However in practice two problems must be addressed. First, BH_3_ reduces the amide protecting groups that are conventionally used with the exocylic amines of cytosine, adenine and guanine. The resulting alkyl amines cannot be removed and therefore represent an undesirable DNA modification. Additionally the trityl cation, which is generated during deprotection of the 5'-hydroxyl, reacts with the phosphite-borane linkage and causes degradation. 

**Scheme 6:**
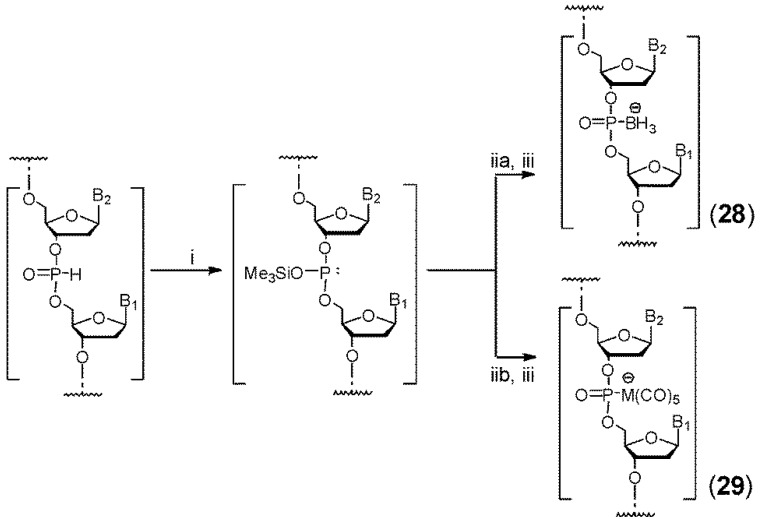
H-Phosphonate method for synthesis of boranephosphonate DNA and metallophosphonate DNA.

The earliest chemical synthesis of boranephosphonate oligomers yielded 2'-deoxyoligothymidines via the H-phosphonate approach (Scheme 6) [38,39,40]. Following synthesis of an oligonucleotide, the H-phosphonate linkages were silylated and the resulting phosphite triesters were boronated to obtain the phosphite triester-borane linkage. The silyl groups were then removed with NH_3_ which generated boranephosphonate-linked 2'-deoxyoligothymidines **28**. Using this approach, 10-14mers were successfully prepared in high yields. As this approach has the advantage of not exposing the P (III)-Lewis Acid complex to multiple rounds of strong acids, it was found to be well suited for preparing metallo phosphonate dinucleotides as well (compound **29**) [33]. In place of borane the silyltriester was treated with pentacarbonyltungstate or pentacarbonylmolybdate.

In order to prepare boranephosphonate linked oligomers containing all four nucleobases, Wada has developed the phosphotriester approach [41,42]. In this method a pre-boronated phosphitylating agent (triethylammonium *bis*(2-cyanoethyl) boranophosphate) was coupled to a 5'-dimethoxytrityl-2'-deoxynucleoside to yield 5'-dimethoxytrityl-3'-boranephosphonate-2'-deoxynucleoside diester. Coupling of this synthon to a 2'-deoxynucleoside attached to a support through the 3'-hydroxyl was carried out using 3-nitro-1, 2, 4-triazol-1-yl-*tris*(pyrrolidin-1-yl)phosphonium hexafluorophosphate as an activator. Triethylsilane was added during acid deprotection in order to scavenge trityl cations. The low step-wise coupling yields however limited the utility of this approach. 

*Reagents*: (i). *N,O*-bistrimethylsilylacetamide/DMF, (iia). BH_3_.THF or BH_3._SMe_2_, (iib). M(CO)_5_.THF, (iii). aq. NH_3_.

We have reported an alternative method, adapted from RNA synthesis, that uses 5'-*O*-silyl 2'-deoxynucleosides 3'-phosphoramidites containing *N*-trimethoxytrityl (TMTr)-protected nucleobases [43]. These protecting groups were found to be stable toward reduction by borane. Thus boronation could be performed after each coupling step to obtain the phosphite triester-borane linkage. Removal of the TMTr groups at the end of the synthesis using aqueous acetic acid did not lead to trityl cation induced degradation of the boranephosphite triester linkages. Mixed base 14mer oligonucleotides containing phosphodiester and boranephosphonate diester linkages could be synthesized in good yields. The same strategy was also useful for the preparation of oligomers containing up to six methylphosphineborane modifications within a 16-mer 2'-deoxyoligonucleotide [31]. For borane-phosphonates as well as methylphosphineboranes, attempts to synthesize longer oligomers however led to many degradation products. These results were perhaps due to the instability of these linkages towards iterative treatment with basic fluoride solutions that were required for removal of 5'-silyl protection. In addition DNA synthesizers had to be equipped to handle basic fluoride. This component of the synthesis procedure therefore limited the broad adoption of the methodology. 

**Scheme 7:**
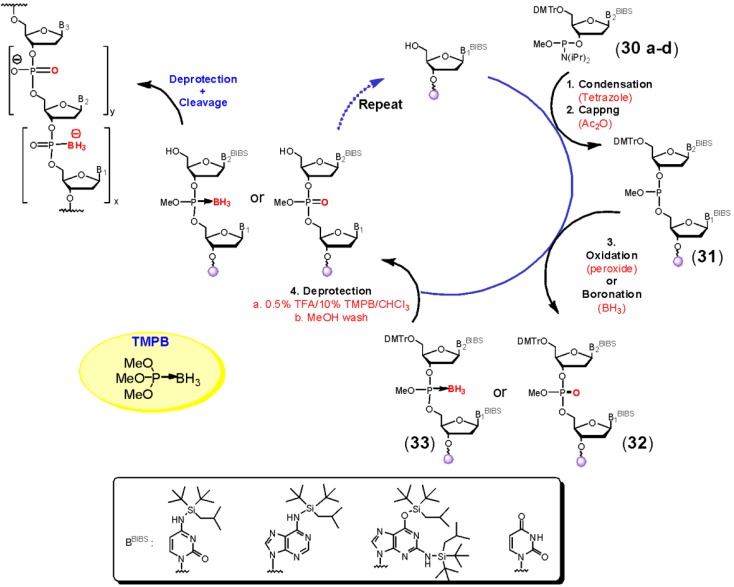
N-Silyl Protected Phosphoramidites as Synthons for bpDNA.

To circumvent these obstacles we have recently developed *N*-silyl protected 5'-dimethoxytrityl-2'-deoxynucleoside 3'-phosphoramidites (**30a**–**c**; Scheme 7) [44]. This silyl group (di-*tert*-butylisobutylsilyl) is stable towards boronation as well as all other reagents used in DNA synthesis. These synthons in conjunction with the use of a more efficient trityl scavenger (trimethylphosphite borane; TMPB) allowed us to prepare 24–30 mer bpDNA oligomers in yields similar to those obtained for standard phosphate linked DNA. Moreover these conditions allow synthesis of bpDNA essentially using the same cycle as described in Figure 2 for standard DNA synthesis. The only modification involves replacement of the iodine oxidation reaction with boronation (Step 3). This leads to conversion of the phosphite triester **31** to a trialkylphosphite-borane derivative **33**. If desired, oxidation of **31** with *tert*-butylhydroperoxide can be performed in order to produce the phosphate triester **32**. Thus oligonucleotides having any desired combination of phosphate and boranephosphonate linkages can be synthesized by this procedure. 

**Scheme 8:**
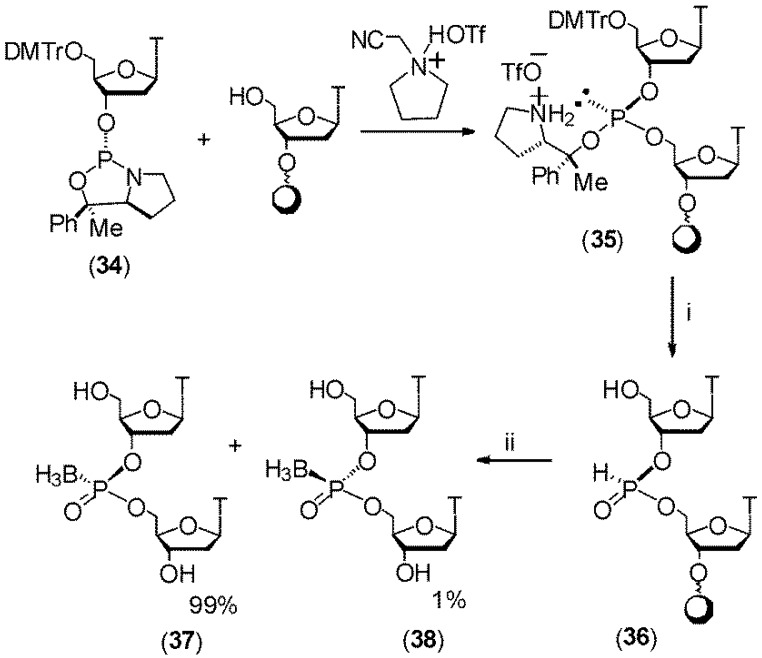
Synthesis of stereodefined boranephosphonate linked DNA.

A future goal is to prepare stereoregular bpDNA and bpRNA oligomers using chemical methods. Such oligomers as demonstrated by the Ramsay-Shaw lab using enzymatically synthesized bpDNA or bpRNA have superior biochemical properties when compared to the diastereomeric mixtures that are currently obtained through chemical synthesis. In this respect a noteworthy report by Iwamoto *et al.* [45] achieved the stereoregular synthesis of boranephosphonate 2'-deoxyoligothymidines using 3'-*O*-oxazaphospholidine derivatives of 2'-deoxythymidine. A pure stereoisomer of this synthon (compound **34**), when activated, generates stereodefined phosphite triesters **35** that can be converted to boranephosphonates **37** or **38** in a stereospecific manner via an H-phosphonate intermediate **36 **(Scheme 8). Taken together these advances raise the possibility of mixed based, stereoregular synthesis of boranephosphonate linked DNA.

*Reagents and Conditions*: (i). 1% Trifluoroacetic acid in CH_2_Cl_2_. (ii). BH_3_.SMe_2_/*N,O*-bistrimethylsilylacetamide/DMF (1:1:8, v/v/v), RT, 15 min; then sat. NH_3_/CH_3_OH, RT, 1 h.

Boranephosphonate diesters posses many properties that are intermediate between P (III) and P (V) compounds. For example they have a ^31^P-NMR chemical shift of 94 ppm, which lies between 140 ppm for phosphite triesters and 0 ppm for phosphates. These compounds are stable to mild acids (pH > 2) and oxidizing agents (e.g., *tert*-butylhydroperoxide) but react with stronger acids or oxidizers such as iodine [46]. The significantly greater stability of borane-P (III) complexes, relative to analogous nitrogen complexes, stems from the fact that, in addition to donation of the lone pair from the P atom, back donation from the σ obital of the BH_3_ group to empty orbitals on the P atom also occurs. The reduced hydridic character of BH_3_ is indirect evidence for this explanation. Substituents on phosphorous also affect the strength of the backbonding interaction [47] and thus the overall stability of the P-B bond. Unpublished experiments from our laboratory demonstrate that replacement of the non-bridging oxygen in boranephosphonate diesters by the less electronegative methyl group significantly increases the hydridic properties of the BH_3_ group. Systematic evaluation of such factors in the future has the potential to create new DNA derivatives with desired chemical and biochemical properties.

The biochemical properties of boranephosphonate linked nucleic acid oligomers have been investigated. They are stable towards hydrolysis by nucleases [36], are effective substrates for RNaseH and siRNA [48], and are more lipophilic than unmodified DNA. Current work in our laboratory has also revealed that these oligomers can be transfected into HeLa cells in the absence of lipid.

We have recently reported that bpDNA reacts with metal ions that possess a high reduction potential (Ag^+^, Au^3+^ and Pt^2+^) [49]. The products of these reductions are metallic nanoparticles. Investigations of this reaction further revealed that the boranephosphonate linkage is converted into a phosphate diester when carried out in water or to a phosphate triester in simple alcohols. We have taken advantage of this combination of reducing properties and an ability to undergo Watson-Crick base pairing to incorporate bpDNA at specific locations within DNA assemblies. This in turn allowed us to carry out deposition of metal nanoparticles onto these assemblies with high spatial resolution [44]. 

## 6. Conclusions

Chemical methods that allow synthesis of DNA/RNA and various derivatives have had tremendous effects on a large number of disciplines. At the heart of all these developments are fundamental discoveries in the chemistry of phosphorous. In particular the reactivity of P (III) compounds led to phosphoramidites as extremely stable, but easily activated synthons for condensation with various nucleophiles to generate DNA or RNA in very high yields. This discovery was a significant milestone, initially in the nucleic acids field, but more recently in other research areas as well. Chemists have also taken advantage of the versatility of phosphorous chemistry in order to create a large number of oligonucleotide derivatives. The majority of these analogs have been developed for use as therapeutics and several such candidates are in advanced clinical trials. In addition DNA derivatives that can combine the binding properties of oligonucleotides with chemically useful functionality are beginning to find applications in new research areas such as nanotechnology and data storage.

The ability to chemically synthesize DNA that is several hundred nucleotides in length is another challenge that awaits chemists. While current methods lead to the synthesis of DNA 20–30 nucleotides in length and more recently to oligomers 150 in length [10] in very high yields and purity, extension to ultra long regimes raises many issues. Thus the field is ripe once again for fresh developments in the chemistry of phosphorous that will allow realization of these goals. 
